# Are pediatric Open Access journals promoting good publication practice? An analysis of author instructions

**DOI:** 10.1186/1471-2431-11-27

**Published:** 2011-04-09

**Authors:** Joerg J Meerpohl, Robert F Wolff, Gerd Antes, Erik von Elm

**Affiliations:** 1 German Cochrane Center, Institute of Medical Biometry and Medical Informatics, University Medical Center Freiburg, Berliner Allee 29, D-79110 Freiburg, Germany; 2 Division of Pediatric Hematology & Oncology, Department of Pediatrics, University Medical Center Freiburg, Mathildenstrasse 1, D-79106 Freiburg, Germany; 3 Kleijnen Systematic Reviews Ltd, Unit 6, Escrick Business Park, Riccall Road, Escrick, York, YO19 6FD, UK; 4 Swiss Paraplegic Research, Guido-A-Zaech-Strasse 4, CH-6207 Nottwil, Switzerland

## Abstract

**Background:**

Several studies analyzed whether conventional journals in general medicine or specialties such as pediatrics endorse recommendations aiming to improve publication practice. Despite evidence showing benefits of these recommendations, the proportion of endorsing journals has been moderate to low and varied considerably for different recommendations. About half of pediatric journals indexed in the Journal Citation Report referred to the Uniform Requirements for Manuscripts of the International Committee of Medical Journal Editors (ICMJE) but only about a quarter recommended registration of trials. We aimed to investigate to what extent pediatric open-access (OA) journals endorse these recommendations. We hypothesized that a high proportion of these journals have adopted recommendations on good publication practice since OA electronic publishing has been associated with a number of editorial innovations aiming at improved access and transparency.

**Methods:**

We identified 41 journals publishing original research in the subject category "Health Sciences, Medicine (General), Pediatrics" of the Directory of Open Access Journals http://www.doaj.org. From the journals' online author instructions we extracted information regarding endorsement of four domains of editorial policy: the Uniform Requirements for Manuscripts, trial registration, disclosure of conflicts of interest and five major reporting guidelines such as the CONSORT (Consolidated Standards of Reporting Trials) statement. Two investigators collected data independently.

**Results:**

The Uniform Requirements were mentioned by 27 (66%) pediatric OA journals. Thirteen (32%) required or recommended trial registration prior to publication of a trial report. Conflict of interest policies were stated by 25 journals (61%). Advice about reporting guidelines was less frequent: CONSORT was referred to by 12 journals (29%) followed by other reporting guidelines (MOOSE, PRISMA or STARD) (8 journals, 20%) and STROBE (3 journals, 7%). The EQUATOR network, a platform of several guideline initiatives, was acknowledged by 4 journals (10%).

Journals published by OA publishing houses gave more guidance than journals published by professional societies or other publishers.

**Conclusions:**

Pediatric OA journals mentioned certain recommendations such as the Uniform Requirements or trial registration more frequently than conventional journals; however, endorsement is still only moderate. Further research should confirm these exploratory findings in other medical fields and should clarify what the motivations and barriers are in implementing such policies.

## Background

Medical research reports and publication practice in biomedicine have been under increased scrutiny over the last decades. Selective reporting of study results and related publication bias has been confirmed in several empirical studies in different disciplines and settings[1]. Further, there is continued concern regarding conflicts of interest that are not disclosed by article authors [2] and cases of scientific misconduct[3]. Non-reporting of study results has been identified also in pediatric research[4]. For instance, significant differences in the risk-benefit profile of selective serotonin re-uptake inhibitors (SSRIs) in children were found in a meta-analysis of studies published in peer-reviewed journals and unpublished data[5]. The data suggesting that SSRIs are linked to an increased risk of suicide or suicidal thoughts had not been published[6].

In the past, several recommendations have been proposed to improve the reporting and publication practice in biomedicine: First, the International Committee of Medical Journal Editors (ICMJE) published the "Uniform Requirements for Manuscripts submitted to Biomedical Journals"[7]. This widely used guideline is currently endorsed by over 700 journals and covers issues such as ethical conduct and reporting of biomedical research, preparation and publishing of manuscripts and editorial policies. Second, the problem of publication bias and selective outcome reporting has been widely analyzed over the last 15 years[1]. Registration of clinical trials and studies of other types prior to patient enrollment has been advocated as an important first step to tackle this problem. Third, authors but also journal editors and reviewers might have financial ties or personal interests in conflict with an article being submitted for publication[8,9]. The debate about this problem has led journals to require disclosure of potential conflicts of interest. Recently, a uniform conflict of interest disclosure form was proposed jointly by major medical journals[10]. Finally, published reporting guidelines such as the CONSORT Statement provide guidance to authors and aim at improving the completeness and accuracy of publications[11,12]. Further, they facilitate the critical appraisal by readers. Endorsement and implementation of these reporting guidelines has been studied for general medicine journals [13,14] but less so for journals in specialties such as pediatrics.

Journals and their editors play a key role in promoting and ensuring transparency in biomedical publishing. Previously, we focused on pediatric journals indexed in the Journal Citation Report and found that the advice given to authors regarding the above mentioned four domains was moderate to low[15]. Reflecting these findings, we wondered whether the low uptake might be due to hesitation or even reluctance of editors of these journals to experiment with editorial procedures.

Open-access electronic publishing has been associated with a number of editorial innovations aiming at improved access to and transparency of research results[16,17]. The new model was a response to the dilemma between increasing prices for journal subscriptions on one side and decreasing resources of academic institutions to finance access to the scientific literature. Open Access publications are generally made available online to anyone anywhere with no charges for access while recovering costs by charging publication fees from authors. Open Access journals usually provide peer review like journals following the conventional publishing model. It has been argued that electronic Open Access publishing does not change significantly content and quality of research articles but improves access to research findings[18].

We wondered whether journals adopting this new publication model take up recommendations which aim to ensure publication of research results in an unbiased and transparent manner. We therefore set out to elucidate the coverage of the four domains Uniform Requirements, trial registration, conflicts of interest and reporting guidelines in Open Access pediatric journals. We then compared our results with findings from "conventional" JCR-indexed pediatric journals analyzed earlier[15].

## Methods

We accessed the Directory of Open Access Journals http://www.doaj.org on 4^th ^of September 2009, identified 43 journals listed in the "Health Sciences - Medicine (General) - Pediatrics" category and extracted information on their start year and publication language. We excluded one journal that does not publish original research articles (Foro Pediátrico) and another journal (Pediatric Cardiology Today) because it was continued as one of the included journals (Congenital Cardiology Today) in 2005. From the websites of the 41 included journals we downloaded the author instructions in September 2009. Two authors (JJM and RFW) read each document and classified information about the geographical location of the main editorial office using the following groups: Africa, Australasia, Europe (without UK), United Kingdom (UK), North America, and South America. Further, we defined three categories of publishers: open access publishing houses, professional societies/academic institutions and other publishers. Using relevant keywords in electronic full text searches we then identified any information on the following four domains of good publication practice:

• Endorsement of the ICMJE Uniform Requirements,[7]

• Requirement of trial registration,

• Editorial policies for disclosure of conflicts of interests,

• Endorsement of five reporting guidelines and related explanatory papers:

○ CONSORT (Consolidated Standards of Reporting Trials) [19,20] and its extensions;

○ STROBE (Strengthening the Reporting of Observational Studies in Epidemiology);[21,22]

○ STARD (Standards for Reporting of Diagnostic Accuracy);[23,24]

○ MOOSE (Meta-analysis of Observational Studies in Epidemiology);[25]

○ QUOROM (Quality of Reporting of Meta-analyses) [26] which has recently been revised and renamed to PRISMA (Preferred Reporting Items for Systematic Reviews and Meta-Analyses)[27,28].

For each of these items, we analyzed the wording of the author instructions and determined whether it was "required" (i.e. a submitted manuscript would not be accepted if the item was not considered) or "recommended" (i.e. its use or fulfillment ought to be considered). Two investigators (JJM, RFW) extracted and categorized information independently. Discrepancies occurred in less than 1% of items and were all resolved by discussion among the investigators.

For comparison, data from a previous study on 69 pediatric journals indexed in the Journal Citation Report (JCR) were used[15].

## Results

The editorial offices of the 41 included journals were located in 21 countries all over the world. Eleven were located in Australasia; fourteen in Europe, nine in South America, six in North America and one in Africa (Table 1). These 41 journals were published by 32 different publishers. Thirteen journals were published by open access publishing houses: BioMed Central (n = 5), Medknow Publications (n = 4), Hindawi Publishing Corporation (n = 2), Libertas Academica (n = 1) and Bentham (n = 1). Fourteen were published either by regional/national professional organisations or academic institutions, and another fourteen by various other publishers. One journal (Indian Pediatrics) was indexed in both the Directory of Open Access Journals and the Journal Citation Report 2008.

**Table 1:** Recommendations provided in author instructions of Open Access (OA) pediatric journals

Recommendation		Geographical location of editorial office No. (% of column total)						Category of publisher No. (% of column total)		
	**All journals** **N = 41**	**Africa** **N = 1**	**Australasia** **N = 11**	**Europe**		**South America** **N = 9**	**North America** **N = 6**	**OA Publishing House** **N = 13**	**Other publisher** **N = 14**	**Professional organization** **N = 14**
									
				**without UK N = 11**	**UK N = 3**					

ICMJE Uniform Requirements for Manuscripts	27 (65.9)	1 (100)	7 (63.6)	7 (63.6)	3 (100)	7 (77.8)	2 (33.3)	10 (77.0)	8 (57.1)	9 (66.3)

Trial registration	13 (31.7)	1 (100)	3 (27.3)	2 (18.2)	3 (100)	3 (33.3)	1 (16.7)	8 (61.5)	1 (7.1)	4 (28.6)

Conflict of interest	26 (63.4)	1 (100)	7 (63.6)	6 (54.5)	3 (100)	6 (66.7)	3 (50)	13 (100)	6 (42.9)	7 (50.0)

CONSORT	12 (29.3)	1 (100)	5 (45.5)	1 (9.1)	3 (100)	1 (11.1)	1 (16.7)	9 (69.2)	0 (0)	3 (21.4)

STROBE	3 (7.3)	1 (100)	2 (18.2)	0 (0)	0 (0)	0 (0)	0 (0)	2 (15.4)	0 (0)	1 (7.1)

STARD	8 (19.5)	1 (100)	3 (27.3)	1 (9.1)	3 (100)	0 (0)	0 (0)	7 (53.8)	0 (0)	1 (7.1)

MOOSE	8 (19.5)	1 (100)	3 (27.3)	1 (9.1)	3 (100)	0 (0)	0 (0)	7 (53.8)	0 (0)	1 (7.1)

PRISMA (QUOROM)	8 (19.5)	1 (100)	3 (27.3)	1 (9.1)	3 (100)	0 (0)	0 (0)	7 (53.8)	0 (0)	1 (7.1)

EQUATOR	4 (9.8)	0 (0)	1 (9.1)	0 (0)	3 (100)	0 (0)	0 (0)	4 (30.8)	0 (0)	0 (0)

ICMJE - International Committee of Medical Journal Editors, CONSORT - Consolidated Standards of Reporting Trials, STROBE - Strengthening the Reporting of Observational Studies in Epidemiology, STARD - Standards of Reporting Diagnostic Accuracy, MOOSE - Meta-analysis of observational studies in epidemiology, PRISMA - Preferred Reporting Items for Systematic Reviews and Meta-Analyses, EQUATOR - Enhancing the Quality and Transparency of Health Research

Only one journal, "Revista Chilena de Pediatria", started publishing in the 1980s. Six journals started in the 1990s, while most of them (n = 34) started publishing in 2000 or later. The majority are published in English (n = 23), while 6 are published in Spanish, 4 in Turkish and 2 in Portuguese. Six journals publish their articles in two or more different languages with all but one publishing in English.

### ICMJE Uniform requirements

The ICMJE Uniform Requirements for Manuscripts were mentioned by 27 journals (66%) (Table 1). Of those, 23 (85%) referred to the web address http://www.icmje.org, where the full document can be downloaded. The Uniform Requirements were most often mentioned in one of the following contexts: (1) the journals support and follow the Uniform Requirements in general, (2) further information on trial registration can be found in the Uniform Requirements and (3) they are recommended as a reference document for manuscript style (e.g. formatting of bibliographies).

### Trial registration

Trial registration was mentioned in thirteen of the 41 journals (32%), out of which nine required and four recommended trial registration prior to publication of a manuscript (Table 1). Several of these journals referred to the ICMJE website for further guidance on trial registration. Two journals did not mention any suitable trial registry in their author instructions.

### Conflict of interest policies

Policies for disclosure of conflicts of interest were found in the author instructions of 26 journals (63%) (Table 1). Six journals stated that they publish information on potential conflicts together with the manuscript. For twenty journals, the author instructions did not specify how the authors' conflicts of interests are handled. The remaining fifteen journals did not provide any information on disclosure of potential conflicts of authors.

### Reporting guidelines

The CONSORT statement was the reporting guideline that was cited most often, i.e. in 12 of 41 journals, 29% (Table 1). Eight journals required authors to follow the CONSORT checklist when preparing manuscripts reporting on trials or to submit a completed checklist together with the manuscript. Four recommended the use of the CONSORT statement when preparing manuscripts. The web address, http://www.consort-statement.org, was given by eleven journals. Each of the reporting guidelines STARD, MOOSE and QUOROM/PRISMA was mentioned in the author instructions of eight journals (20%). The STROBE statement was mentioned by three journals (7%). The EQUATOR network http://www.equator-network.org, an initiative collating several reporting guideline, was mentioned by four journals (10%).

### Analysis according to geographical location and category of publisher

First, the analysis according to geographical location of editorial office showed some variation in the guidance given: the three journals with an editorial office in the UK gave the most guidance while journals based in North America provided much less guidance (Table 1).

Second, we wondered whether the type of publisher influenced the amount of guidance given to authors and therefore analysed our findings according to category of publisher. The group of journals published by Open Access publishers (n = 13) offered the most guidance to their authors: the Uniform Requirements were mentioned by ten journals (77%), while trial registration was required or recommended by eight (62%). All thirteen journals described a conflict of interest policy on their website. The CONSORT statement was mentioned by nine journals (69%), while STARD, MOOSE and PRISMA were referred to by seven journals (54%). The EQUATOR network was mentioned by four journals (31%) (Table 1). Recommendations regarding the four domains of good publication practice were less often mentioned by journals published by professional societies or academic institutions and by other publishers.

## Discussion

We analyzed to what extent author instructions of Open Access pediatric journals reflect recommendations on four domains of editorial policy: Uniform Requirements, trial registration, conflicts of interest and reporting guidelines. The uptake of recommendations regarding these domains was moderate and varied considerably across journals. The proportion of Open Access journals giving advice in these four domains was slightly higher as compared to 69 journals listed in the Journal Citation Report 2008, except for conflict of interest policies (Table 2)[15].

**Table 2:** Recommendations provided in author instructions of 69 pediatric journals indexed in the Journal Citation Report [15]

Recommendation	No. of journals giving recommendation (%) (N = 69)
ICMJE Uniform Requirements for Manuscripts	38 (55)

Trial registration	16 (23)

Conflict of interest	54 (78)

CONSORT	14 (20)

STROBE	3 (4)

STARD	4 (6)

MOOSE	3 (4)

PRISMA (QUOROM)	4 (6)

ICMJE - International Committee of Medical Journal Editors, CONSORT - Consolidated Standards of Reporting Trials, STROBE - Strengthening the Reporting of Observational Studies in Epidemiology, STARD - Standards of Reporting Diagnostic Accuracy, MOOSE - Meta-analysis of observational studies in epidemiology, PRISMA - Preferred Reporting Items for Systematic Reviews and Meta-Analyses

The interpretation of these data and the comparison with our previous survey of author instructions [15] need to consider several aspects. First, the number of journals examined was not very large with 41 Open Access journals and 69 journals indexed in the Journal Citation Report. However, both selections did not represent samples drawn from a larger group but an analysis of all journals meeting the entry criteria. Second, clustering of journals that are run by the same publisher might have influenced our results. We aimed to address this by analysing the data by type of publisher. Third, our analysis was based on the policies as documented in the online instructions that potential authors would consult as first reference. We did not determine whether the editorial staff of the journals applies additional procedures that are not reflected in the author instructions e.g. to encourage or even enforce adherence of authors to the promoted policies. Such additional procedures could only be identified by surveying editorial staff to elucidate their motivations to implement new policies or any barriers to do so. However, journal procedures were not the interest of our current study. Finally, our study is of exploratory nature. We are not aware of any other studies on author instructions of Open Access journals or comparisons with journals with another publishing model. One might speculate that the situation is similar in journals in general medicine or other specialties. Additional studies including other than pediatric journals should be undertaken to determine whether our results can be generalized to other Open Access journals.

Several of the included Open Access journals are more recent than the journals that are indexed in the Journal Citation Report. When they set up their author instructions they might have sought guidance on the most recent developments in good publication practice and consequently included recommendations on the four domains investigated in this survey from the start.

Although the empirical evidence on improvement of publication practice due to endorsement of these recommendations is still limited, providing such recommendations in the author instructions might be considered a means to enhance reporting quality by newly founded or less well-established journals[16,29].

Several of the included journals were run by publishers dedicated to Open Access publishing. This group of journals offered the most extensive guidance to their authors. It is conceivable that these publishers provided a master copy of author instructions to be adapted by the journal's editorial team. This circumstance might have contributed to the higher proportion of guidance that is offered by these OA journals.

Finally, our analysis of author guidelines could be complemented by an assessment of the content published by the Open Access journals. Previous empirical studies have looked at the quality of published articles, in particular before and after introduction of reporting guidelines, but did not focus on different publishing models[11,30].

## Conclusions

Pediatric OA journals are a heterogeneous group of journals ranging from journals run by national societies to new publications launched by dedicated OA publishers. Overall, they do give at least as much guidance to authors as do conventional pediatric journals. Interestingly, differences exist between types of publishers. However, the uptake of good publication practices could still be improved in all groups. Whether these results can be generalized to Open Access journals in other specialties or in general medicine needs to be investigated. Reasons for differences should be elucidated e.g. by surveys of journals editors.

## Competing interests

Erik von Elm is one of the authors of the STROBE Statement and Academic Editor of PLoS ONE, an Open Access journal not included in this study. The other authors are not aware of any potential conflicts of interest.

## Authors' contributions

JJM and RFW conceived the study, developed the data extraction form, extracted the data and performed the data analysis. They also drafted the manuscript. GA helped with coordination of this project and critically discussed the manuscript. EvE was involved with data analysis, interpretation and provided statistical advice. He also was involved in writing the manuscript. All authors read and approved the final manuscript.

## Pre-publication history

The pre-publication history for this paper can be accessed here:

http://www.biomedcentral.com/1471-2431/11/27/prepub
